# Divide and conquer: broadly neutralizing antibody combinations for improved HIV-1 viral coverage

**DOI:** 10.1097/COH.0000000000000800

**Published:** 2023-05-19

**Authors:** Kshitij Wagh, Michael S. Seaman

**Affiliations:** a Theoretical Division, Los Alamos National Laboratory and New Mexico Consortium, Los Alamos, New Mexico; b Center for Virology and Vaccine Research, Beth Israel Deaconess Medical Center, Harvard Medical School, Boston, Massachusetts, USA

**Keywords:** Broadly neutralizing antibodies, clinical trials, HIV-1, passive immunization, prevention, therapy

## Abstract

Successful HIV-1 prevention and therapy will require broad and potent coverage of within-host and global viral diversity. Broadly neutralizing antibody (bNAb) combination and multispecific therapeutics provide an opportunity to meet this challenge due to the complementary activity of individual antibody components. Here, we review the principles and applications of this concept.

**Recent findings:**

The Antibody Mediated Prevention (AMP) trials have demonstrated the high bar for neutralization potency and breadth that bNAb-mediated prevention modalities will need to achieve to have a meaningful impact on the HIV-1 epidemic. Additional clinical studies have recently shown that an even higher bar may be required for therapeutic inhibition of the diverse within-host quasispecies present in viremic and aviremic people with HIV-1 (PWH). We discuss how the complementarity of bNAbs in terms of neutralization profiles, resistance mutations and coverage of within-host quasispecies may overcome these stringent requirements and lead to effective bNAb combination or multispecific antibody based prophylactic and therapeutic strategies.

**Summary:**

The design of next-generation bNAb-based combination or multispecific therapeutics for the prevention and/or treatment of HIV-1 infection will need to leverage the complementarity of component bNAbs to maximize the potency and breadth that will be required for clinical success.

## INTRODUCTION

**FIGURE 1:**
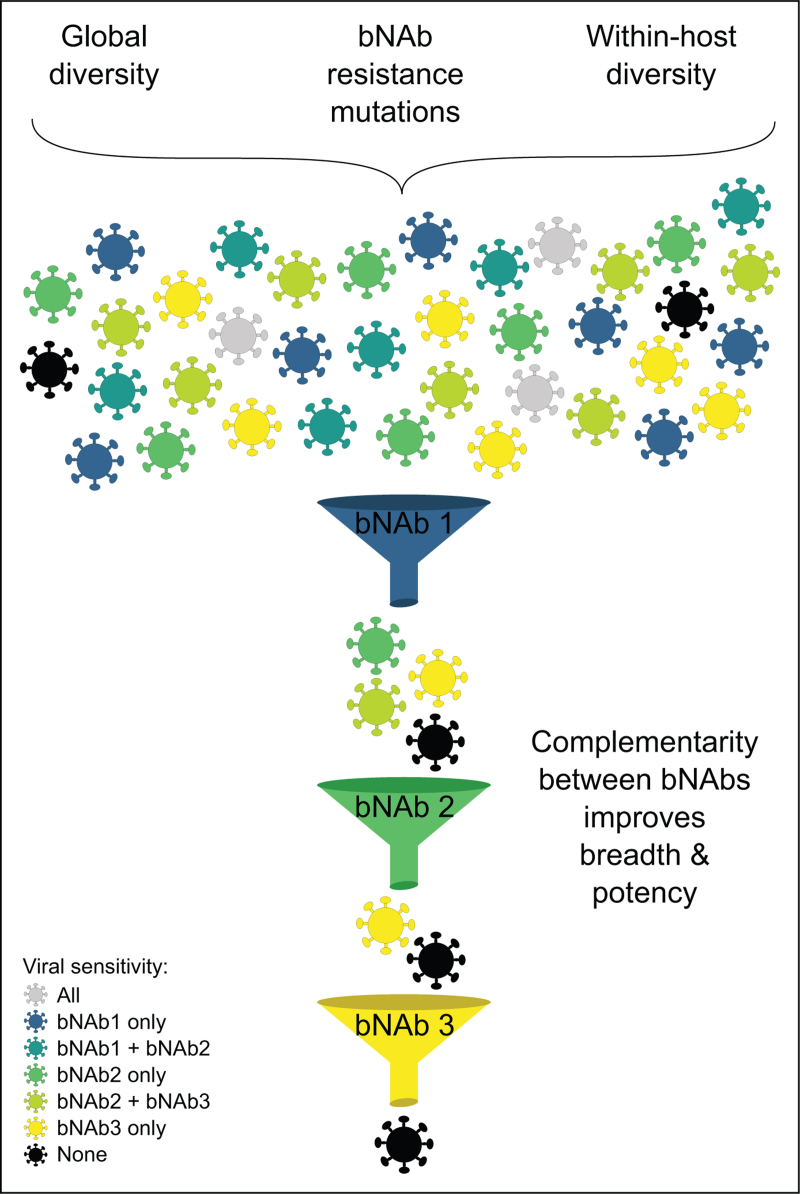
Multiple levels of complementarity between bNAbs. Viral diversity in terms of bNAb sensitivity or resistance originates from global diversity of circulating HIV-1 strains, bNAb-resistance mutations and the within-host diversity of HIV-1 quasispecies in chronically infected PWH. This diversity is schematically depicted by different colored virions that exhibit different levels of sensitivity/resistance to each of the three bNAbs depicted as shown in the legend on bottom left. The complementarity between bNAbs is shown as independent sieves that filter out (i.e. neutralize) different viruses sensitive to one or more of the bNAbs. This results in improved potency and breadth of neutralization by bNAb combinations. bNAb, broadly neutralizing antibody.

Over the past decade, improvements in antibody isolation techniques have yielded many highly potent and broad anti-HIV neutralizing antibodies [1]. Such broadly neutralizing antibodies (bNAbs) isolated from different people with HIV-1 (PWH) target recurring epitopes on the Env glycoprotein such as the CD4 binding site (CD4bs), V2-apex, V3-glycan, the membrane-proximal region (MPER), gp120-gp41 interface and the silent face of gp120 (see [2] for a recent review). Intense study of bNAbs has not only guided HIV-1 vaccine design efforts [3], but has also promoted their use in passive immunization strategies for the prevention and/or treatment of HIV-1 infection [2,4,5]. The latter focus has come of age with a flurry of clinical studies that together suggest that the requirements for potent and broad neutralization to have meaningful clinical impact are much higher than anticipated. While it has been known that bNAb combinations can improve neutralization potency and breadth (see e.g. [6]), the stringent requirements highlighted by recent clinical studies underscore the necessity of using combination approaches. Critical questions of how many and which bNAbs to use in combinations have been discussed before [2,4,7]. The key principle underlying this choice is how bNAbs targeting different Env epitopes provide complementary neutralization coverage in multiple scenarios from *in vitro* neutralization to targeting within-host diversity *in vivo* (Fig. 1). Here, we review this complementarity in bNAb neutralization profiles from multiple vantage points that will help inform the design of next-generation combination and multispecific bNAb prophylactic and therapeutic modalities. 

**Box 1:**
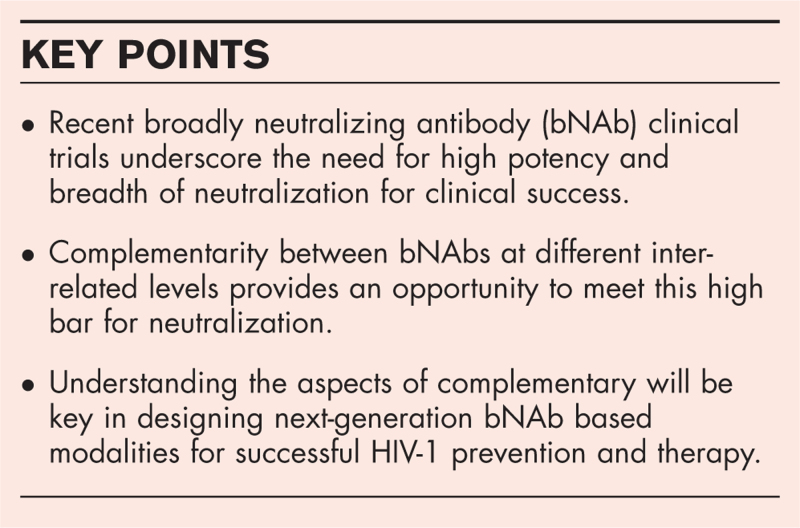
no caption available

## STRINGENT NEUTRALIZATION REQUIREMENTS FOR CLINICAL SUCCESS OF bNAb BASED PREVENTION AND THERAPEUTIC STRATEGIES

Several recent clinical trials studying bNAb-based prevention and therapeutic strategies have underscored the high bar of neutralization potency and breadth that will be required for success *in vivo*. First, the landmark phase 2 Antibody Mediated Prevention trials (AMP; Clinicaltrials.gov identifiers: NCT02716675 and NCT02568215) tested the efficacy of a single bNAb targeting the CD4bs (VRC01) to prevent HIV-1 infection in populations of high-risk women and men who have sex with men [8]. Although these trials did not observe significant reduction of HIV-1 acquisition, subsequent analyses using pseudotype assays did validate the sensitivity of the infecting virus to neutralization by VRC01 *in vitro* as a correlate of protection [9^▪▪^]. To achieve ≥90% protection, predicted serum ID_80_ titers (PT_80_) of ≥ 200 would be required, which translates to VRC01 neutralizing titers of 80% inhibitory concentration (IC_80_) ≤0.1 μg/ml against infecting viral isolates assuming average pharmacokinetic concentration of VRC01 from the 30 mg/kg dose group in the AMP trials [9^▪▪^,^10^]. Second, two recent phase 1 clinical trials have explored the therapeutic efficacy of a dual bNAb combination of 3BNC117 (anti-CD4bs) and 10–1074 (anti-V3-glycan) following 3–7 bNAb infusions to assess viral control in HIV-1 viremic participants or aviremic participants undergoing analytical treatment interruption (ATI) (NCT03526848 [11^▪^] and NCT03571204 [12^▪^]). While this dual bNAb combination provided encouraging signs of prolonging the time to viral rebound following ATI, rebound viruses exhibiting resistance to 10–1074 emerged in most participants [11^▪^]. It is not known whether such 10–1074 resistant viruses were preexisting in the latent reservoir or evolved *de novo*, but this is alarming regardless since it suggests a high propensity of resistance may exist in people living with HIV to at least some individual bNAbs. In the setting of 3BNC117 and 10–1074 combination therapy in viremic participants, this phenomenon was even more accentuated; most participants had viral loads return to near baseline levels following transient declines post-bNAb infusions, and the rebound viral population exhibited resistance to both bNAbs [12^▪^]. However, it should be noted that in this small study, 3 out of 5 viremic participants had preinfusion baseline viruses that were resistant to one of the two bNAbs.

This phenomenon was further highlighted by a third phase 1 clinical trial that evaluated viremic control after a single dose of a triple bNAb combination of VRC07–523LS (anti-CD4bs), PGT121 (anti-V3-glycan) and PGDM1400 (anti-V2-apex) (NCT03205917) [13^▪^]. In this study, all viremic participants showed recovery of viral load between 7–20 weeks postinfusion, despite two out of three participants having sensitive viruses at baseline to each of the three bNAbs. At rebound, neutralization resistance to PGT121 and PGDM1400 had developed in all participants, while only modest resistance (six-fold reduction in IC_80_ titers) to VRC07–523LS was found in a single participant. A combined analysis of this study together with prior studies involving the dual combination of 3BNC117 and 10–1074 and monotherapy with either 3BNC117 or VRC01 suggested that on average PT_80_ titers of >1000 would be needed to control viremia (or more precisely to avoid viremic rebound), which would translate to single or combination neutralizing titers of IC_80_ < 0.02 μg/ml using the above average pharmacokinetic concentration from the AMP trials. Together, these clinical studies outline that potent neutralization of virtually all circulating strains of HIV-1 will be required for clinical success. Although to date no single bNAb can meet this stringent requirement, combinations of bNAbs or multispecific antibodies have the potential to do so [9^▪▪^,^14^,15^▪^].

## COMPLEMENTARITY OF *IN VITRO* NEUTRALIZATION PROFILES OF BROADLY NEUTRALIZING ANTIBODIES

**FIGURE 2:**
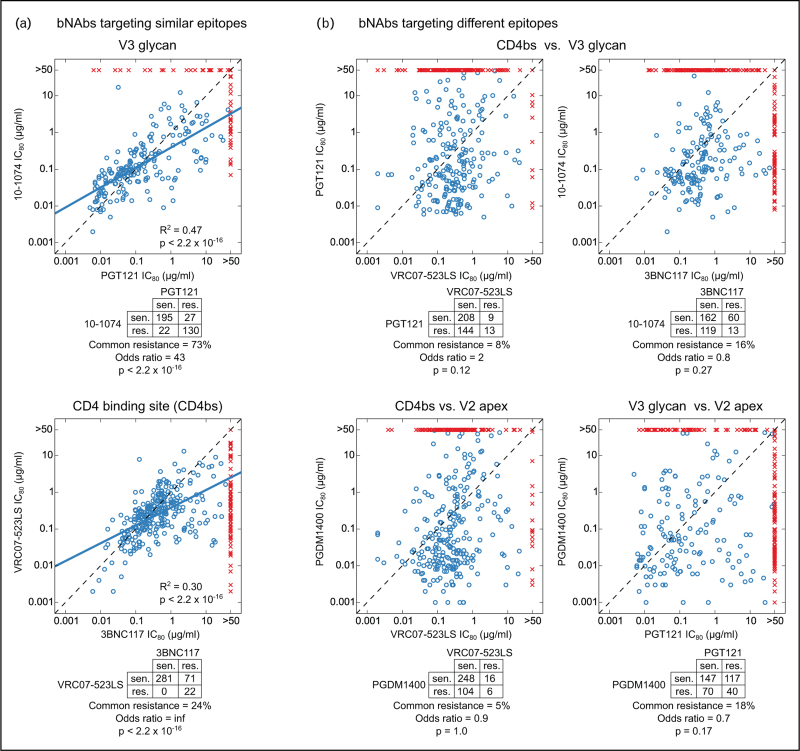
Complementarity in neutralization by bNAbs targeting different epitopes. (a) Correlated neutralization between bNAbs that target similar epitopes. Top and bottom panels show two distinct bNAbs targeting either V3-glycan epitope or CD4bs epitope, respectively. Each point in the scatter plot shows IC_80_ titers for the two bNAbs against the same virus. Blue circles indicate viruses sensitive to both bNAbs (IC_80_ < 50 μg/ml), while red crosses indicate viruses resistant to one or both bNAbs (IC_80_ > 50 μg/ml). Dotted lines indicate identity, and thick blue lines indicate the linear regression trend line between log_10_ IC_80_ titers using only the viruses sensitive to both bNAbs. Square of the Pearson correlation coefficient (*R*^2^) and the corresponding *P*-value is indicated. The contingency table below each scatter plot shows the number of viruses sensitive to both bNAbs (top left), resistant to either bNAb but sensitive to the other (off-diagonal) and resistant to both bNAbs (bottom right). The percentage of viruses that are resistant to both bNAbs from the number of viruses resistant to either bNAb is reported, along with the odds ratio and Fisher's exact test *P*-values. (b) Uncorrelated neutralization between bNAbs targeting different epitopes. Same format as panel (a), except neutralizing titers of bNAbs targeting different epitopes are analyzed. In contrast to bNAbs targeting similar epitopes in (a), bNAbs targeting different epitopes in (b) do not show significant correlation in IC_80_ titers (*R*^2^ = 0.02–0.07, thus linear regression trends are not shown) and have no significant overlap in sensitive/resistant viruses. Neutralization data was obtained from CATNAP on the Los Alamos HIV Database (www.hiv.lanl.gov) and comes from different studies, as previously described [7]. bNAb, broadly neutralizing antibody.

It is well established that combinations of two or more bNAbs targeting different epitopes can significantly improve potency and breadth of *in vitro* neutralization against genetically diverse global isolates compared to each component bNAb alone [6,14]. Two primary reasons can explain this improvement. First, individual bNAbs targeting different epitopes demonstrate complementary neutralization propensities against global isolates (Fig. 2). Although bNAbs targeting similar Env epitopes have significantly correlated IC_80_ titers and significant overlap in commonly sensitive or commonly resistant viruses (Fig. 2a), bNAbs targeting different epitopes show uncorrelated IC_80_ titers and no significant overlaps in mutually sensitive or resistant viruses (Fig. 2b). This implies that most viruses that are poorly neutralized by or are resistant to a single bNAb can be potently neutralized by a different bNAb targeting a distinct epitope. Secondly, we have shown that accurate modeling of neutralization by bNAb combinations as a function of neutralization by individual bNAbs relies on the assumption that antibodies targeting distinct epitopes essentially operate independently in combination (“Bliss-Hill model” from ref. [6]). These properties combine to provide much improved potency and especially breadth for bNAb combinations as compared to single component bNAbs, thus improving the probability of meeting the stringent requirements for positive clinical impact.

This complementarity in neutralization breadth and potency profiles of different bNAbs has been leveraged for the design of engineered multispecific antibodies that provide an impressive alternative to bNAb combinations and have the potential to address issues relating to variable pharmacokinetic profiles and challenges associated with the manufacture and administration of multiple bNAbs. While combining the Fabs of two or three different bNAbs in a single multispecific is not novel, with two of the most promising molecules having entered clinical testing (10E8.4/iMAb (NCT03875209) [16] and N6/PGDM1400-10E8v4 or SAR441236 (NCT03705169) [17,18^▪^]), newer approaches in multispecific antibody design have shown promise [19,20^▪^]. In particular, the multabody format utilizing a self-assembling ferretin nanoparticle has demonstrated significant synergy through combining multiple bNAb specificities that improve avidity as well as neutralization breadth and potency when tested against a large panel of genetically diverse HIV-1 Env pseudoviruses. Multabody molecules can further incorporate Fc domains able to elicit nonneutralizing antibody effector functions, and have also demonstrated favorable pharmacokinetic profiles in preliminary mouse experiments [20^▪^].

## COMPLEMENTARITY OF BROADLY NEUTRALIZING ANTIBODY RESISTANCE MUTATIONS

bNAbs isolated from multiple PWH have been found to recurrently target distinct epitopes on the Env trimer, with CD4bs, V2-apex and V3-glycan (“high mannose patch”) epitopes among the most frequently targeted [3,21]. It is expected that bNAbs targeting distinct epitopes have different Env resistance mutations since such mutations, which disrupt biochemical antibody–epitope interactions, will typically be located on the corresponding bNAb epitope. Indeed, a systematic study of statistically robust Env mutations that are associated with bNAb neutralization resistance has shown that bNAbs targeting different epitopes have distinct resistance “signatures”, in contrast to bNAbs targeting the same epitope class where resistance signatures are typically shared [22]; this is despite finding statistically robust outside of epitope signatures in addition to those within-epitope for each bNAb. Because each viral isolate can have a diverse constellation of sensitive, resistant or neutral amino acids to each bNAb class, bNAbs targeting different epitope-classes will thus exhibit complementary neutralization sensitivity/resistance profiles when tested against large representative panels of HIV-1 pseudovirus isolates (Fig. 2b).

The independence of bNAb-resistance mutations has further been demonstrated in clinical studies of bNAb combinations in which the evolution of resistance mutations to one bNAb did not confer resistance to other bNAbs in the combination, and multiple independent resistance mutations were required to confer resistance to multiple bNAbs. For example, three out of four viremic participants treated with the dual combination of 3BNC117 and 10–1074 developed neutralization resistance only to 10–1074 [23], and rebound viruses from all 3 participants in the triple bNAb combination trial developed resistance to PGT121 and PGDM1400 by acquiring independent resistance mutations in each respective epitope [13^▪^].

Another potential mechanism for partial bNAb resistance can be posttranslational modifications on Env such as glycosylation heterogeneity or conformational heterogeneity in genetically identical virions. This can result in a fraction of clonal virions exhibiting resistance to bNAbs, and is often observed as incomplete neutralization curve plateaus or persistent fractions [6,24,25^▪^]. We have shown that combining bNAbs can overcome such heterogeneous resistant fractions of virus [6], suggesting that bNAbs are complementary also in the impact of posttranslational modifications underlying partial bNAb resistance.

## COMPLEMENTARITY OF bNAbs FOR COVERING WITHIN-HOST DIVERSITY

The challenges that must be overcome for clinical success of bNAb-based prevention and therapeutic regimens are further amplified by the issue of HIV-1 diversity that arises from both the heterogeneity of within-host quasispecies in each person living with HIV-1 as well as regional differences in the genetic makeup of locally circulating strains (e.g. regional clade-specific predominance, although each clade also has substantial diversity within) [7]. Not only should bNAb modalities be able to contend with this immense multiscale genetic diversity, but they must also do so at the high *in vivo* potency requirements as outlined above.

Passive immunization studies in PWH have demonstrated that resistance to any single bNAb is readily observed in the viral quasispecies at baseline (i.e. before bNAb therapy), even with the relatively low number of participants enrolled in these trials. Across the 3BNC117 and 10–1074 combination trials described above, 13 out of 34 participants had fully resistant viruses at baseline to one or both bNAbs [11^▪^,12^▪^]. In the triple bNAb combination trial, two out of three participants had fully resistant viruses at baseline to either one or two of the bNAbs [13^▪^]. The high propensity of preexisting bNAb resistance in the within-host quasispecies arises from the high rate of Env evolution that occurs during chronic infection under selection pressures from humoral immune responses. This problem can be exacerbated for PWH who develop antibodies targeting similar epitopes as therapeutic bNAbs [21], where viral escape from within-host responses could lead to partial or full resistance to therapeutic bNAbs. Particularly impacted are certain bNAb classes, such as those targeting V2-apex and V3-glycan epitopes, where complete neutralization escape can be achieved by single amino acid/glycan mutation, while relatively lesser impact is observed for classes like CD4bs targeting bNAbs that tend to require a more complex mutational profile to mediate partial or complete resistance [11^▪^,12^▪^,13^▪^]. Furthermore, some specific HIV-1 subtypes can be fully or partially resistant to certain bNAb classes (an extreme example is the uniform CRF01 resistance for V3-glycan bNAbs) [7], thus leading to geographic biases in prevalence of bNAb resistance.

Although individual bNAb resistance is commonplace, resistance to multiple bNAbs may be more rare. Only two participants in the dual bNAb combination trials [11^▪^,12^▪^] and only one participant in the triple bNAb combination trial had baseline viruses fully resistant to two bNAbs, and no participant showed full resistance to all three bNAbs [13^▪^]. This follows from the fact that resistance to a bNAb combination will involve resistance to each bNAb in the combination, each of which requires different resistance mutations. Thus, on an average more mutations will be required for resistance to bNAb combinations than for individual bNAbs, and hence, the probability of evolving resistance mutations to multiple bNAb classes in the viral quasispecies is lower. A further barrier to simultaneously evolve escape at multiple bNAb epitopes could be the higher associated fitness costs [15^▪^,^26^,^27^]. This improved coverage of within-host diversity across hosts provides another facet of bNAb complementarity.

The advantage of redundant bNAb coverage, i.e. multiple bNAbs in the combination simultaneously covering within-host diversity, in therapeutic settings is clearly demonstrated by combination bNAb clinical trials, where participants harboring baseline viruses potently neutralized by two or three bNAbs typically exhibit prolonged viral suppression or viral control as compared to those participants with viruses sensitive to only a single bNAb [11^▪^,12^▪^,13^▪^]. However, such redundant active bNAb coverage will also be useful in the setting of HIV-1 prevention as it will improve the coverage of within-host diversity of chronic donors in transmission events. It is impossible to extensively sample the quasispecies of chronically infected PWH at the population level, and representative pseudovirus panels designed to capture global diversity typically only include a single virus from each sampled host, thus resulting in a lack of detailed information about their diverse quasispecies. In this scenario, we have advocated the use of multiple active bNAb coverage, i.e. the fraction of viruses neutralized simultaneously by multiple bNAbs, to rank bNAb combinations [6]. This follows from the above logic that if a particular virus from a given host is neutralized by multiple bNAbs, the chance of this host harboring other viruses that are resistant to each bNAb is lower. Using globally representative pseudovirus panels, we have found that substantial dual active bNAb coverage is difficult to attain for even the best two bNAb combinations, and to achieve high dual active bNAb coverage requires combinations of at least 3 bNAbs or a bispecific bNAb combined with a conventional bNAb [14].

## CONCLUSION

Recent clinical studies have underscored the challenges associated with the use of single or combination bNAbs for the prevention and/or treatment of HIV-1 infection. To address these stringent requirements, the different levels of complementarity among bNAbs for improved potency and breadth need to be carefully considered in the design and development of combination strategies that can overcome the barriers for clinical success. While in this review we have focused on conventional bNAb neutralization effector activity, the same principles of bNAb complementarity will also apply for inhibition of cell-cell spread and nonneutralizing Fc-mediated effector functions.

## Acknowledgements

*None.*

### Financial support and sponsorship

*Bill & Melinda Gates Foundation Collaboration for AIDS Vaccine Discovery (INV-036842).*

### Conflicts of interest

*There are no conflicts of interest.*

## REFERENCES AND RECOMMENDED READING

Papers of particular interest, published within the annual period of review, have been highlighted as:

▪ of special interest
▪▪ of outstanding interest
